# Forty Years in the Making: Understanding the Molecular Mechanism of Peptide Regulation in Bacterial Development

**DOI:** 10.1371/journal.pbio.1001516

**Published:** 2013-03-19

**Authors:** Marta Perego

**Affiliations:** Department of Molecular and Experimental Medicine, The Scripps Research Institute, La Jolla, California, United States of America

## Abstract

Signal transduction systems are influenced by positive and negative forces resulting in an output reflecting the sum of the opposing forces. The Rap family of regulatory protein modules control the output of two-component signal transduction systems through protein∶protein and protein∶peptide interactions. These modules and their peptide regulators are found in complex signaling pathways, including the bacterial developmental pathway to sporulation, competence, and protease secretion. Two articles published in the current issue of *PLOS Biology* reveal by means of crystallographic analyses how the Rap proteins of bacilli are regulated by their inhibitor Phr peptide and provide a mechanistic explanation for a genetic phenotype isolated decades earlier. The Rap-Phr module of bacterial regulators was the prototype of a family that now extends to other bacterial signaling proteins that involve the use of the tetratricopeptide repeat structural fold. The results invite speculation regarding the potential exploitation of this module as a molecular tool for applications in therapeutic design and biotechnology.

Cell signaling by oligopeptides is a critical component of the biology of eukaryotic and prokaryotic cells. In microorganisms such as Gram-positive bacteria, small peptides have been found to regulate a variety of cellular functions, providing the bacteria with the ability to communicate and change behavior of the same or of other species in response to conditions and perturbations of the environment [1]. Studies in the spore-forming model organism *Bacillus subtilis* were among the first to identify pathways in which peptide signaling played a regulatory role.

## Forty Years of History

In 1991 the phosphorelay signal transduction system was discovered as the pathway controlling the initiation of the sporulation process that bacilli undertake when growth conditions become unfavorable. The novelty of the discovery was in the multicomponent nature of the system, with four functionally distinct components, in contrast to the two-component structure of the bacterial signal transduction systems described up to that point [2].

**Figure 1:**
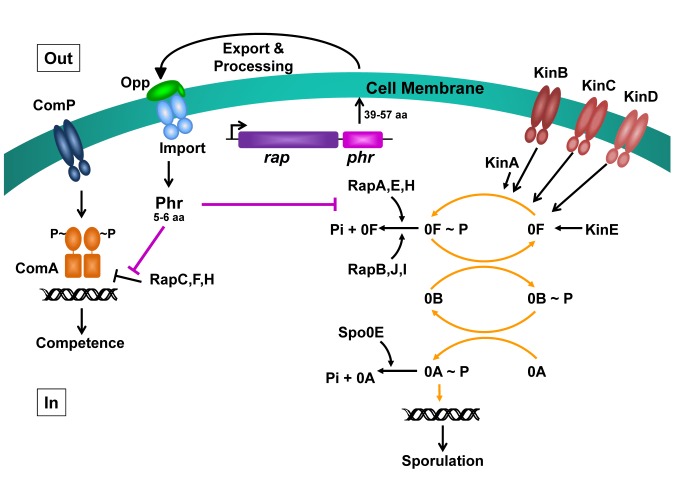
Schematic representation of *Bacillus subtilis* signaling pathways regulated by Rap-Phr modules. The *rap* and *phr* genes are often genetically associated and co-transcribed. The products of the *rap* genes are known to either dephosphorylate the Spo0F∼P (0F∼P) intermediate component of the phosphorelay controlling sporulation initiation (RapA,B,E,H,I,J; right-hand side) or to inhibit the DNA-binding activity of the ComA response regulator and transcription factor for competence development (RapC,F,H; left-hand side) or of the DegU response regulator for protease production (RapG; not depicted). The phosphorelay is activated by five histidine sensor kinases (KinA and KinE are cytoplasmic while KinB, KinC, and KinD are membrane bound). Spo0F∼P transfers the phosphoryl group to the Spo0B phosphotransferase, which in turn transfers it to the Spo0A response regulator and transcription factor. Spo0A∼P activates transcription of the genes required for sporulation initiation. Spo0A∼P is subject to dephosphorylation by the members of the Spo0E family of phosphatases. Alternative pathways to sporulation induce transcription of *rap* genes whose products dephosphorylate Spo0F and block the process: growth induces expression of RapB while competence induces RapA and RapH through the ComP-activated ComA response regulator (the transcription regulatory network is not depicted). ComP is a membrane-bound histidine sensor kinase. The products of the *B. subtilis phr* genes, which vary in length between 39 and 57 amino acids, are first secreted outside the cell and then re-imported by the oligopeptide permease system (Opp) following processing events that result in Phr peptides five to six amino acids long. Once internalized, each Phr peptide interacts with and inhibits its paired Rap protein so that sporulation or competence can develop (for reviews, see [14],[15],[40],[41]).

The complexity of the phosphorelay was deemed justified by the complexity of the sporulation process itself that, at some point, is irreversible and thus is initiated only if no alternative survival avenues are available. This rationale was the basis for hypothesizing that each component of the phosphorelay could represent an entry point for regulatory mechanisms of survival alternative to sporulation. This hypothesis was strengthened by the discovery of two families of aspartyl-phosphate phosphatases, the Spo0E and Rap families, that targeted the Spo0A∼P and the Spo0F∼P response regulators of the phosphorelay, respectively (Figure 1) [3]–[5]. While mechanisms regulating Spo0E protein activity or gene transcription remain largely unknown, studies on the first two members of the Rap family, RapA and RapB, revealed an intriguing regulatory complexity.

First, the genes encoding RapA and RapB were found to be transcriptionally controlled by growth conditions antithetical to sporulation, i.e., competence to DNA transformation and exponential growth, consistent with their role in redirecting cell fate [3],[6]. Then, a mechanism for regulating RapA function was discovered when a deletion in the small open reading frame that follows the *rapA* gene was generated and a sporulation-deficient phenotype was observed, in contrast with the hypersporulation phenotype caused by the deletion of the *rapA* gene [7]. This experiment revealed the existence of the PhrA inhibitor, a five amino acid peptide resulting from a pathway involving the secretion of the pro-peptide product of the *phrA* gene (44 amino acids long) and its re-importation after processing [8],[9]. Once internalized, the PhrA pentapeptide was found able to directly interact with and inhibit RapA phosphatase activity [10]–[12].

## Greater Complexity Emerges

Sequencing of the *B. subtilis* chromosome revealed nine more *rap* genes, seven of which were associated with a linked *phr* gene [13]–[15]. Genetic and biochemical analyses showed that while some, such as RapE and RapJ, were also phosphatases of Spo0F∼P [16],[17], others had different biochemical functions. Both RapC and RapF were found able to inhibit the DNA-binding activity of the ComA competence factor for DNA transformation [18],[19]. Similarly, RapG affected the same activity of DegU regulating extracellular protease production [20]. Additionally, RapH exhibited double specificity by both dephosphorylating Spo0F∼P and inhibiting ComA [21].

Rap proteins share a high level of sequence homology, ∼45%, suggesting the overall structural fold of all is similar; yet there is wide diversity of structurally different targets to which Rap proteins bind and affect function.

Structural predictions were consistent with a two-domain structure and indicated that the C-terminal domain of Rap proteins was organized in tetratricopeptide repeats (TPR), a structural module commonly described in eukaryotic proteins and well known to be involved in protein∶protein and protein∶peptide interactions [15],[22]. The major unsolved questions prior to structural characterization were how Rap proteins attained specificity for their target, what domains were involved in binding the various targets, where did the inhibitory peptides bind, and how were they able to displace the target proteins? Structural studies were necessary to answer these questions and complement the genetic and biochemical work done so far. Structural characterization of Rap proteins' interaction with their target response regulator or their inhibitory peptide has now been accomplished.

## Structural Insights

Two independent reports in the current issue of *PLOS Biology* describe the structural mechanism of Phr binding to Rap proteins and, together with previous reports of the structure of Rap proteins in complex with either ComA or Spo0F, provide the answers raised by the previous genetic and biochemical work [17],[23].

Using crystallographic analyses, the structure of RapF, both unbound and in complex with its inhibitor peptide PhrF (QRGMI), was solved by Gallego del Sol and Marina [24], while the structure of RapJ in complex with PhrC (ERGMT) is reported by Parashar et al. [25], who also report the structure of the RapI protein in the unbound state. The striking revelation is that the binding of the Phr peptide results in a major conformational change in the Rap protein that was unanticipated from studies on TPR-containing proteins so far available.

**Figure 2:**
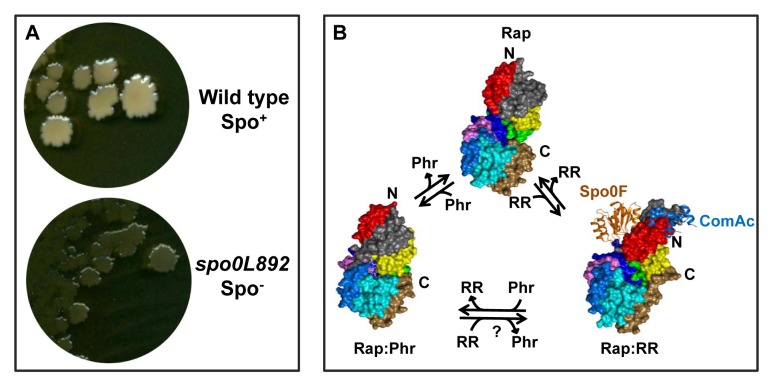
Forty years in the making: from the phenotype of a genetic mutant to its understanding using protein crystals. The *spo0L892* mutant was isolated in the early 1970s (J.A. Hoch, unpublished data) (A) and was characterized by an early sporulation phenotype (thin and transparent colonies versus the thick and opaque colonies of the wild type strain). In the 1990s the gene identified by the *spo0L892* allele was cloned, characterized biochemically, and renamed RapA as the first member of the Rap family. Now, crystal structures have been solved (B) of Rap proteins in complex with their inhibitor peptide (Rap:Phr), or with their target response regulator (Rap:RR; Spo0F is in orange and the C-terminal binding domain of ComA is in blue) or in the unbound form (Rap), providing the molecular explanation for the phenotype of the original *spo0L* mutant. Notably, the ability of Phr peptides to displace the response regulator is proven, but whether the opposite occurs is unknown (?). The N-terminal domain of Rap proteins that undergoes a major relocation upon binding of the peptide is shown in red and grey (compare the position of the red and grey areas in the Rap:Phr and Rap:RR structures, bearing in mind that the C-terminal TPR domains of the two images are shown in identical orientations).

Rap proteins were found to be organized into two modular domains: an N-terminal three-helix bundle connected by a flexible helical linker to the C-terminal domain containing six canonical and one non-canonical TPR domains [17]. The structure of Rap proteins showed that the N-terminal domain interacts with the response regulator and the specificity for the target is the result of the development of two distinct non-overlapping surfaces interacting with either the response regulator fold of Spo0F or the helix-turn-helix DNA-binding domain of ComA [17],[23],[26]. The C-terminal TPR domain is involved with the binding of the peptide inhibitor. Peptide binding induces a constriction of this domain that propagates to the N-terminal three-helix bundle, which is rotated through about 180° while the linking region rotates by about 135° compared to the response regulator-bound Rap protein; this generates two helix-turn-helix structures that pack against the C-terminal domain. As a result, the entire protein has now become one single domain made of nine TPR-like folds (Figure 2B).

Consistent with the biochemical observation that the Phr peptide can displace the response regulator from the preformed complex with its Rap protein [11], the conformational change induced by Phr binding results in a steric impairment of the response regulator binding surfaces on the N-terminal domain. Given the dramatic conformational changes it undergoes upon interaction with the Phr peptide, the unbound Rap protein probably occupies a dynamic equilibrium state between the open conformation (without the peptide and available for interaction with the response regulator) and the closed conformation (bound to the peptide and unavailable for response regulator binding). Crystallographic observations are consistent with this hypothesis and invite dynamic studies by nuclear magnetic resonance approaches.

Overall, the structural data are consistent with previous genetic and biochemical experiments and with original observations reported with the first released structures of TPR domain-ligand peptide complexes [11],[26],[27]. In order for a Rap protein to interact with a response regulator, a domain was likely to fold in a manner reminiscent of the four-helix bundle of the Spo0B phosphotransferase of the phosphorelay or of the two-component system histidine kinases [28],[29]. Indeed, this is the case for the portion of the N-terminal domain of Rap proteins that interacts with Spo0F and the position and orientation of the catalytic Q47 residue of RapH required for dephosphorylation of Spo0F∼P is similar to the position and orientation of the phosphorylatable histidine (His30) of Spo0B that also dephosphorylates Spo0F∼P [17],[26]. Unanticipated, however, was the development by Rap proteins of a distinct interface with a DNA-like conformation for interaction with the DNA-binding domain of ComA [23].

## Rap-Phr Binding: An Issue of Specificity

The binding of PhrF to RapF and PhrC to RapJ shows the use of the same interaction platform within the TPR domain. This platform was first described with the structures of Hop TPR domains bound to their chaperone ligands, the Hsp70 heptapeptide or the Hsp90 pentapeptide [27]. Notably, other TPR-containing proteins use different binding sites to interact with their ligands [30]. The Phr peptides bind in an extended conformation that allows maximal surface exposure, thus promoting interactions with sufficient affinity despite the limited length of the peptides. The interactions include sequence-independent ones with the peptide backbone (anchoring interactions), specific ones with the peptide side chains (specificity determinants), and those involving the C-terminal carboxyl and N-terminal amino moieties of the peptide, likely to ensure proper peptide:TPR domain docking.

The residues involved in anchoring the Phr peptide are understandably conserved within the Rap family, but one, Asn227 of RapF or Asn225 of RapJ, deserves special mention for being a conserved feature of prokaryotic and some eukaryotic TPR domains [26]. This residue makes hydrogen bond(s) with the main chain of the fourth residue of Phr (Met in both PhrF and PhrC) that is, nevertheless, quite variable in the Phr family.

Among the anchoring interactions, of particular interest is the role of a highly conserved aspartate residue (D194 in RapF and D192 in RapJ) that, rather than interacting with the peptide backbone, establishes a side-chain:side-chain salt bridge with the second residue of the pentapeptide, an arginine highly conserved among Phr peptides. This aspartate is the site of the *spo0L892* mutation, an Asp to Asn change at position 194, that originally identified the *spo0L* locus, later renamed *rapA*, and rendered RapA insensitive to PhrA, thus conferring a sporulation-deficient phenotype (Figure 2A) [3],[31].

Comparison of the structural interactions that may be involved with specificity of Rap proteins for the Phr peptides indicates that both complexes—RapF-PhrF and RapJ-PhrC—employ essentially the same residue pairings. Alignment of Rap and Phr amino acid sequences indicates that residue pairs may have coevolved, as some pairing patterns could be inferred, though the number of Rap and Phr sequences available is insufficient to carry out computational studies. However, some differences are noticeable between the two structural complexes in the strength and number of the bonds established. This is likely due to the fact that while the RapF:PhrF interaction is genetically established and physiologically relevant, and therefore likely to represent a best-fit case, the RapJ-PhrC may be artifactual and thus not necessarily optimal.

Because specificity is the result of a combination of factors (residue type, hydrophobicity, charge, and electrostatics), in the case of the Rap proteins that are modulated by a small peptide the question arises as to whether specificity is absolute or relative. In the pure growth condition of the laboratory test tube a limited number of Phr-like peptides are produced, creating the conditions for almost absolute specificity of a given Phr peptide for its genetically associated Rap protein. However, cross talk between unpaired Rap-Phr proteins or interference can be artificially achieved by manipulating peptide concentrations (see RapB-PhrC or RapJ-PhrC) or single amino acid changes [10],[12]. Thus, the myriad peptides present in the bacterial natural environment (theoretically 20^5^ for a peptide of length 5), particularly in the mammalian host of a pathogen, could interfere with the physiology of microorganisms capable of importing them. Comparison of interaction kinetics could be used to define the determinants of specificity between Rap proteins and Phr peptides and may be exploited for the design of molecules that could modulate bacterial physiology for medical or industrial applications [32]. Conversely, given the distribution of TPR-containing proteins in eukaryotic cells, bacterial peptides like the Phr of pathogenic bacilli could exert signaling functions in the mammalian host [33],[34].

## Future Perspectives

The Rap-Phr module of bacterial regulators was the prototype of the RNPP protein family [35] that initially seemed to be restricted to the genus *Bacillus*. Knowledge acquired from the Rap-Phr systems allowed the identification of the analogous PapR peptide that activates the PlcR virulence transcription regulator and the necrotrophic transcription factor NprR with its associated peptide NprX from the *Bacillus cereus/thuringiensis/anthracis* group [35]–[37]. Structural analysis then suggested that the PrgX protein of *Enterococcus faecalis* (with its ligand cCF10) is also a member of this family [35],[38]. Structural predictions now include the Rgg proteins of streptococci with their associated Shp peptides, which would rename the family RRNPP [39]. The RRNPP proteins have in common being modulated by a small peptide (causing either inhibition as in Rap proteins and PrgX or activation as in PlcR, NprR, and Rgg) and being structurally organized in canonical or non-canonical TPR domains. Structures of Rap proteins, PlcR, and PrgX in complex with their ligands also show the use of the same interaction platform with a key asparagine (RapFAsn227 or RapJAsn225 mentioned above) essential for peptide anchoring.

Nevertheless, the structural effect induced by each peptide to the corresponding target protein is quite different. As described in the reports highlighted here, a major conformational change is induced in the N-terminal domain of Rap proteins upon binding of the Phr peptides that prevents binding of the target but does not affect the quaternary structure. Instead, PapR binding to PlcR triggers polymerization that facilitates DNA binding while interaction of PrgX with cCF10 disrupts its tetramerization, weakening its DNA-binding activity [35],[38]. Whether NprX and Shp induce quaternary structural changes in NprR and Rgg as in PlcR and PrgX may be anticipated, but studies are needed to resolve this issue.

On the one hand, the remarkable work of the Marina and Neiditch laboratories bring an end to a research chapter initiated some 40 years ago by providing the molecular mechanism behind a genetic phenotype. On the other hand, however, it opens a new chapter of possible applications in the field of Gram-positive bacteria (characterized by organisms of medical and industrial relevance), and beyond. The adaptation of the TPR fold to a variety of functions invites speculation regarding their exploitation as molecular tools: protein engineering could redesign modules and specificities for a variety of applications in therapeutics and biotechnology. Time will tell whether this story will also have a happy ending.

**Glossary**

**Signal transduction system**: the mechanism used by living cells to transmit information after sensing a given signal. In these systems the presence of a signal is generally transformed into a chemical entity (a phosphoryl group) and transferred from a sensing molecule to an effector molecule.

**Histidine sensor kinase**: one of the two components and the sensing molecule in the bacterial two-component signal transduction systems. It autophosphorylates on an intracellular histidine residue when activated by an extracellular signal specific to each kinase.

**Response regulator**: the second component of two-component systems, it is phosphorylated on an aspartate residue by the paired histidine kinase. It is generally activated once phosphorylated and becomes a transcription factor that controls the expression of the genes required for the response to the signal received by the kinase.

**Phosphorelay**: a signal transduction system where the phosphoryl group is relayed from the sensing molecule (a histidine sensor kinase) to the effector molecule (e.g., Spo0A transcription factor) via two additional intervening molecules (e.g., Spo0F and Spo0B). Originally discovered as the system for sporulation initiation in *B. subtilis*.

**Oligopeptide permease**: a membrane-bound transport system that is involved in the transport through the membrane of peptides, including the Phr peptides of *B. subtilis*.

**Sporulation**: the process undertaken by spore-forming microorganisms (such as bacilli and clostridia) involving development from living and dividing organisms into dormant spores highly resistant to unfavorable conditions.

## Abbreviations

TPR: tetratricopeptide repeat
